# Reversal of Compromised Bond Strength of Bleached Enamel Using Cranberry Extract as an Antioxidant: an In Vitro Study

**DOI:** 10.7759/cureus.6188

**Published:** 2019-11-18

**Authors:** Anusha Eggula, Pranitha V, Dwijendra K.S, Nagarjuna G, Naseemoon Shaik, Mohammadi Fatima

**Affiliations:** 1 Pedodontics & Preventive Dentistry, MNR Dental College and Hospital, Hyderabad, IND

**Keywords:** bleaching, hydrogen peroxide gel, 10% sodium ascorbate, 6% cranberry solution

## Abstract

Background and Objectives: Bleaching reduces the bond strength of enamel, if adhesive restorations are carried out immediately. Reversal of compromised bond strength of bleached enamel by application of antioxidant agents has been reported in the literature. The aim of the study is to assess the neutralizing effect of 6% cranberry (CB) solution on the bond strength of bleached enamel compared with that of 10% sodium ascorbate (SA) solution.

Materials and Methods: Enamel surfaces of 64 extracted human premolar teeth were randomly divided into four groups based on the antioxidant used. Further subgrouping was done in Groups II, III, and IV dividing each group into subgroup A and subgroup B of eight teeth each based on whether the bonding was performed immediately or after a delay of 14 days postbleaching respectively. Shear bond strength (SBS) of the specimens was measured using a Universal testing machine. The data were then tabulated and statistically analyzed using one-way ANOVA (analysis of variance) and Tukey’s post-hoc parametric tests. A p-value of <0.05 was considered statistically significant.

Results: The SBS measurements were compared among the four groups including the subgroups. The SBS was highest in the SA delayed bonding group and lowest in the bleached immediate bonding group.

Conclusion: A 10% SA solution has proven superior to 6% CB solution in the reversal of compromised bond strength following bleaching.

## Introduction

Tooth bleaching has become one of the most popular esthetic dental treatments and also done to enhance a person’s smile. It has been reported that the adhesion between the tooth surface and the bonding agent is hampered postbleaching [1-2]. The contributing factor for this reduced bond strength has been attributed to the oxygen free radicals accountable for the whitening effect; they adversely influence the penetration of the bonding agent into the tooth surface and inhibit the complete polymerization of the bonding agent [3].

Methods proposed to reverse the compromised bond strength of the resin material to bleached enamel are delay of 24 h to four weeks [4], removal of superficial layer of enamel [5], treatment of the bleached enamel with alcohol before the restoration [6], use of adhesives containing organic solvents [7], application of antioxidant agents [3, 8] before bonding the resin composite, and use of antioxidant incorporated bleaching agents [9].

The effect of cranberry (CB) as an antioxidant on bleached enamel has not been investigated so far. Hence, the present study is conducted with the aim to evaluate and compare the effects of 10% sodium ascorbate (SA) versus 6% CB solution on the reversal of compromised bond strength of bleached enamel.

## Materials and methods

**Figure 1:**
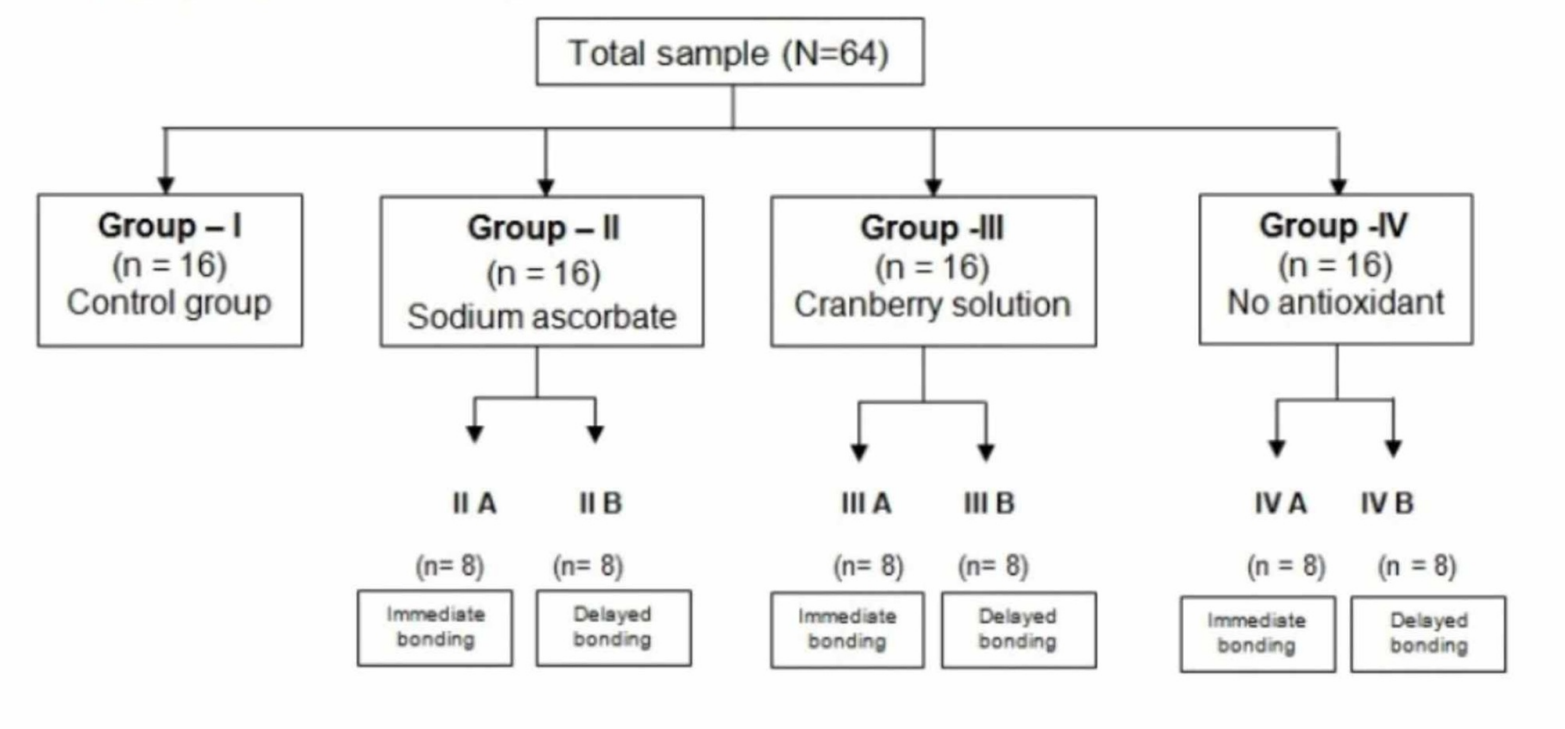
Experimental design.

It is an in vitro study conducted at MNR Dental College and Hospital, Sangareddy, India. Some 64 sound extracted human premolar teeth with intact enamel surfaces without pretreatment with chemicals and devoid of developmental defects were included in the study. Fractured teeth and teeth with dental caries, attrition, abrasion, erosion, and previously restored or endodontically treated teeth were excluded from the study. The teeth were cleaned to remove any residual tissue tags. The roots were amputated from the crowns at the cementoenamel junction using slow speed diamond disc under copious water spray. The teeth were then mounted in cold-cure acrylic resin using Teflon mold, with the buccal surface facing upwards. Some 16 teeth were sampled as Group I (n=16) which served as the positive control group and did not receive any bleaching treatment. Remaining 48 teeth were equally divided into three groups (Groups II, III, and IV) based on the type of antioxidant used after bleaching. The groups were further divided into subgroups A (immediate bonding) and B (delayed bonding after two weeks) based on the storage period before composite build-up. Each subgroup consisted of eight teeth (Figure 1).

Bleaching procedure

**Figure 2:**
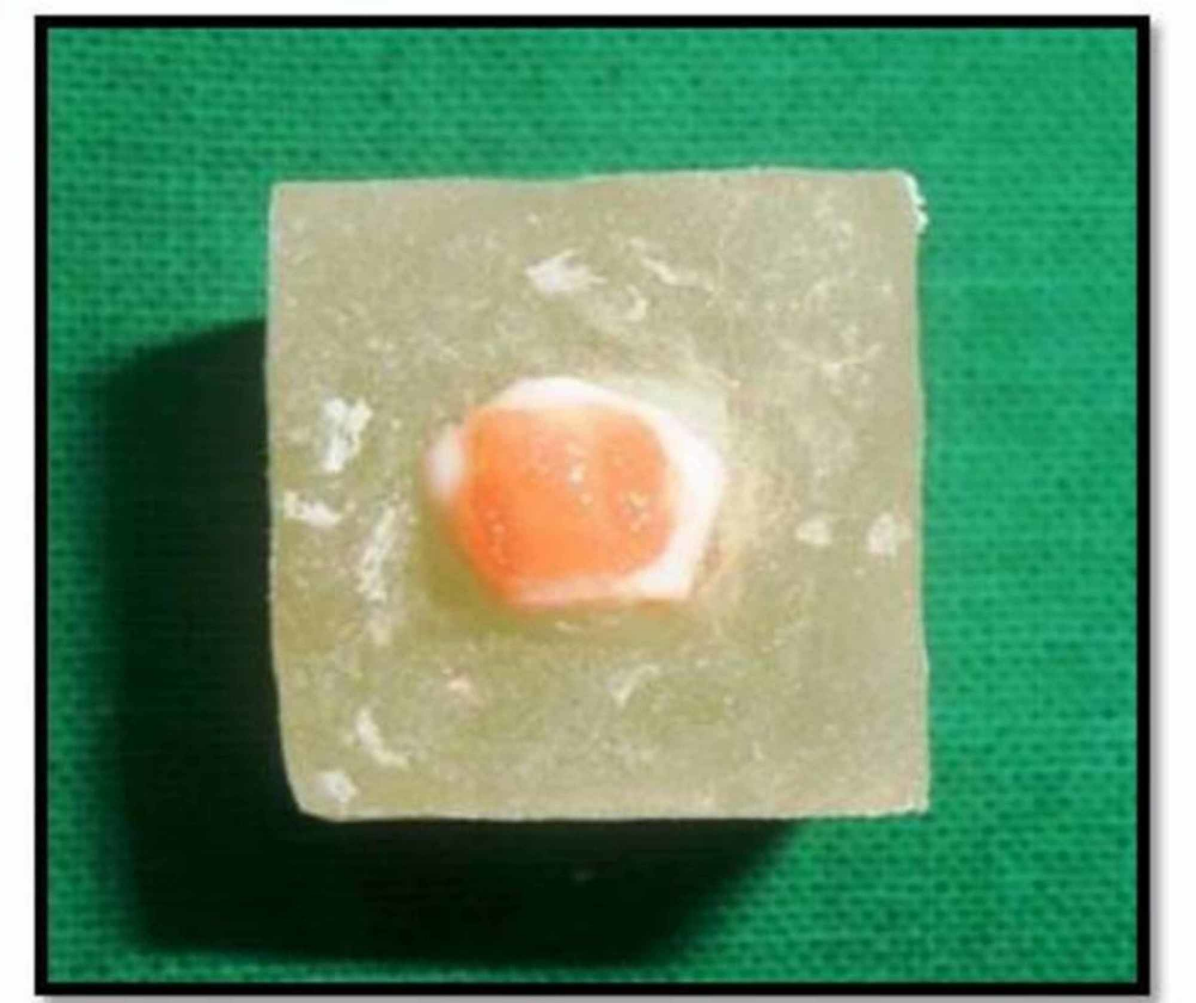
Application of 40% hydrogen peroxide gel.

Bleaching gel containing 40% hydrogen peroxide (Opalescence boost, Ultradent products, Inc, South Jordan UT) was placed on the enamel surface of sample for 20 min according to the manufacturer’s instructions (Figure 2). The bleaching gel was then completely rinsed off with water.

Preparation of solutions

Two solutions were prepared for this study:

1) 10 g of SA (Limcee, Abbott healthcare Pvt. Ltd, India) was dissolved in 100 mL of distilled water to make 10% SA solution.

2) 6 g of CB extract (TracfreeTM, Zydusnutriva, Cadila healthcare Ltd., India) in the form of powder was collected from the tablets and dissolved in 100 mL of distilled water to make 6% CB extract solution.

**Figure 3:**
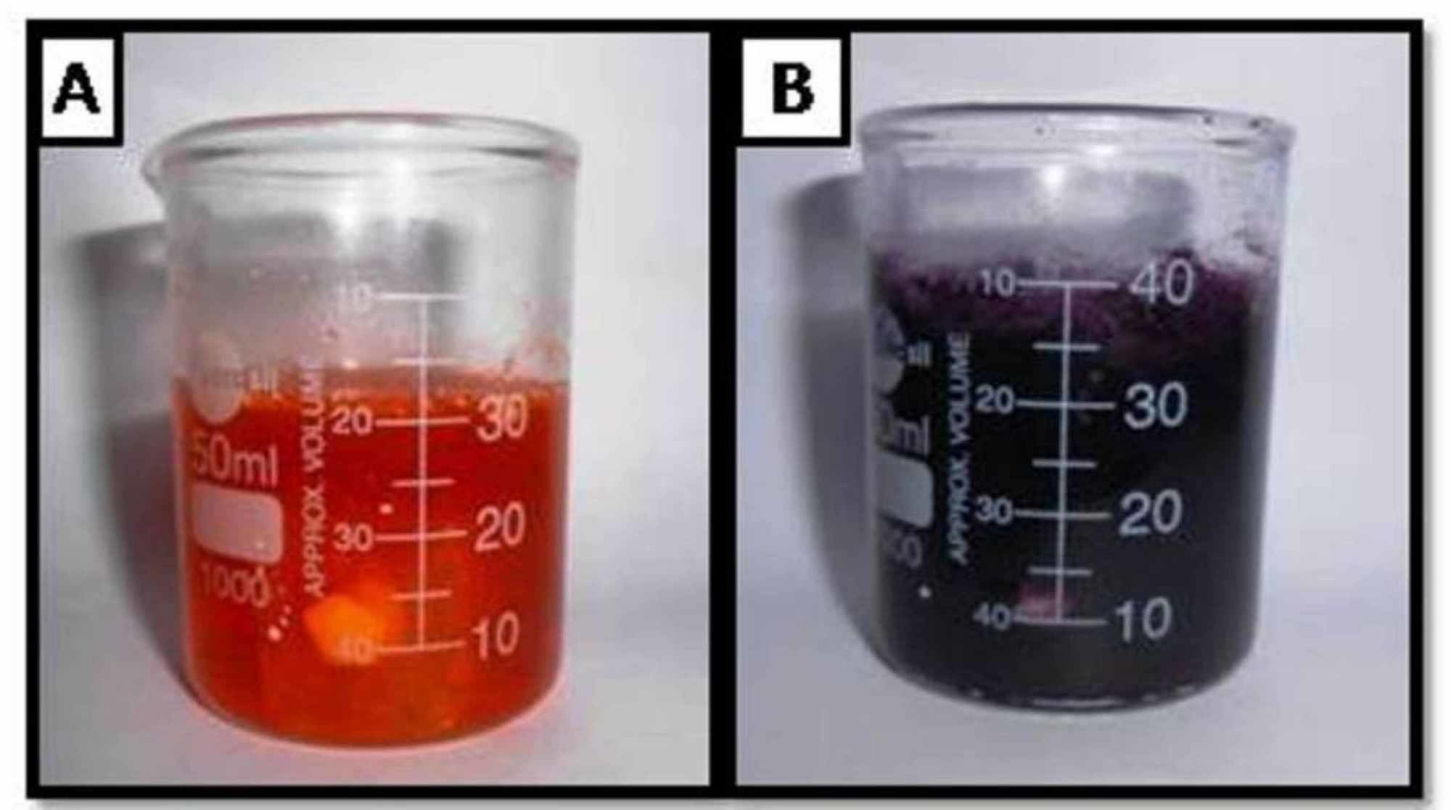
A. Immersion of bleached specimens in 10% sodium ascorbate solution. B. Immersion of bleached specimens in 6% cranberry solution.

The respective samples were immersed in 10% SA and 6% CB extract solution for 10 min (Figure 3A,B) following the bleaching process and then the specimens were rinsed with distilled water and dried with compressed air.

Bonding procedure

All of the specimens were etched with 37% phosphoric acid gel (D-tech Dental technologies, Pune, India) for 15 s and then rinsed with water for 20 s and air dried for 5 s. Adhesive (Prime &Bond®NT Nano-technology dental adhesive, Dentsply, USA) was then applied, gently air thinned, and light cured for 10 s.

A split-mold metal casing (3 mm in diameter and 5 mm in height) was used to maintain uniformity in composite build-up. The casing was placed on adhesive-applied enamel surfaces; composite (Filtek Z350, 3M ESPE, Dental Products, India) was then filled into the hole in layers and each layer was light cured according to the manufacturer’s instructions. After polymerization, mold was removed and specimens were placed in distilled water for 24 h.

**Figure 4:**
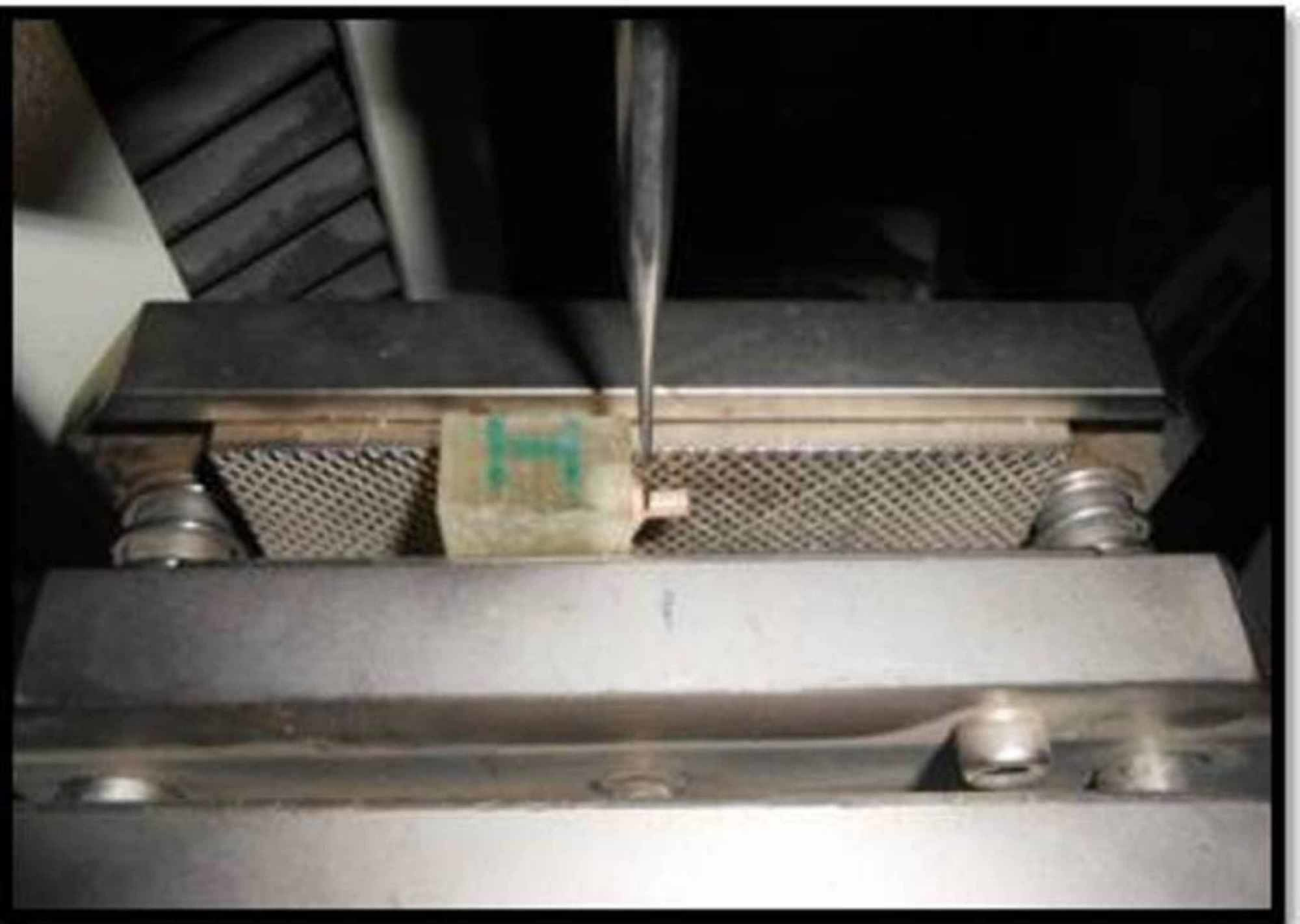
Shear bond strength testing by Universal testing machine.

After 24 hours all specimens were tested for shear bond strength (SBS) in shear mode (using a steel knife edge shearing rod) using a Universal testing machine (Dak System Inc.’s U.T.M, India) at a crosshead speed of 0.5 mm/min (Figure 4). SBS values were expressed in mega pascal (MPa).

The data were subjected to parametric tests -- one way ANOVA (analysis of variance) for multiple group comparison of SBS followed by Tukey’s post-hoc test for group wise comparison depending on the normality of data and p-value (<0.05) was considered statistically significant.

## Results

**Figure 5:**
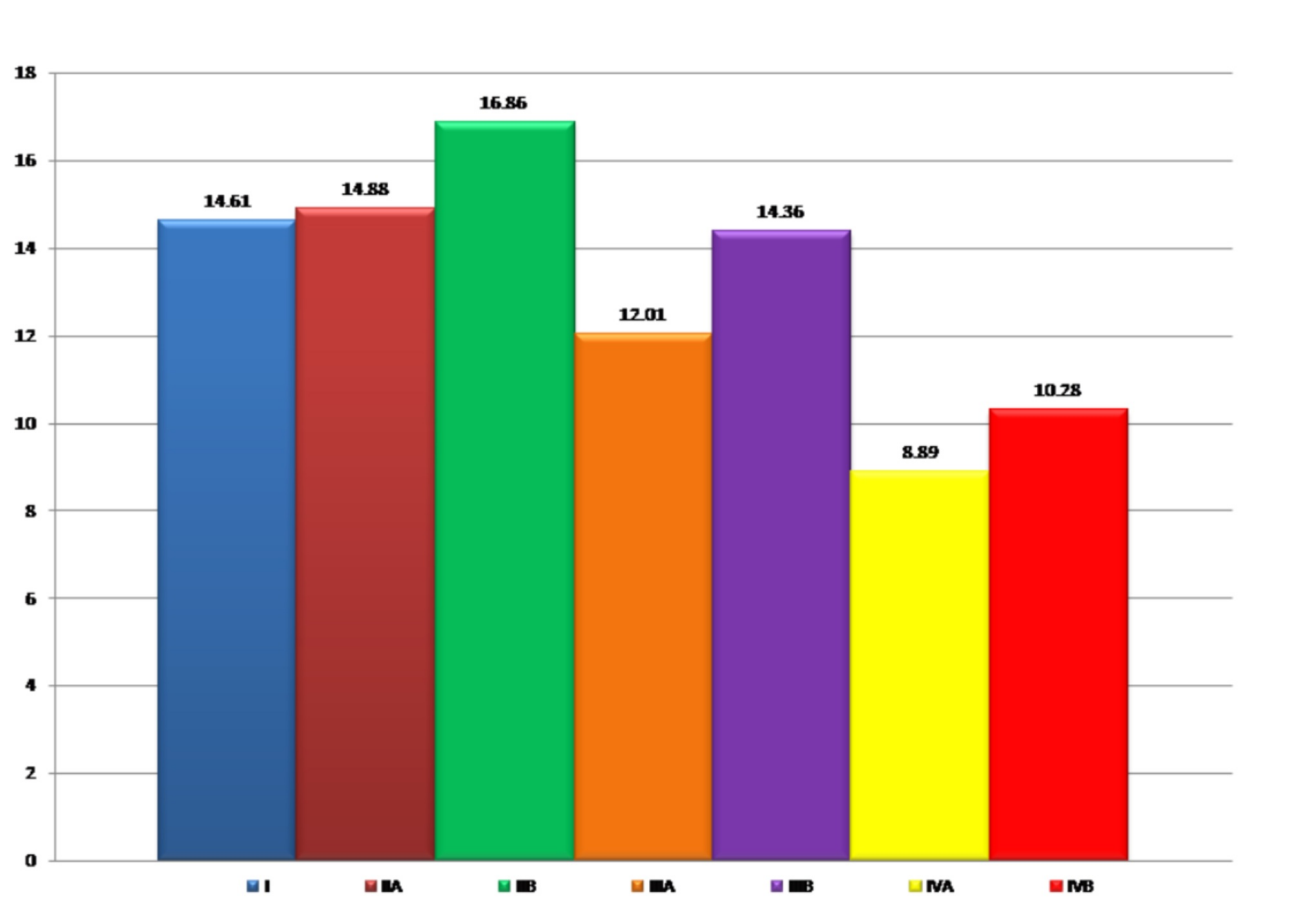
Inter-group comparison of mean shear bond strength (MPa).

The SBS measurements were compared among the four groups including the subgroups and are shown in Figure 5.

The SBS was highest in the SA delayed bonding group and lowest in the bleached immediate bonding group. A statistically significant difference was found between the control group and the bleached group (p=0.006). Among the subgroups of A, Group IIA showed the highest mean SBS value, in the order of Group IIA (Bleaching+10%SA+Immediate bonding) > IIIA (Bleaching+6% CB+Immediate bonding) > IVA (Bleaching+Immediate bonding). Among the subgroups B, Group IIB had the highest SBS; in the order of Group IIB (Bleaching+10% SA+delayed bonding) > Group IIIB (Bleaching+6% CB+Delayed bonding) > Group IVB (Bleaching+Delayed bonding) (Table 1). In the inter-group comparison, the highest mean value of SBS was recorded for Group IIB in the order of IIB > IIA > I > IIIB > IIIA > IVB > IVA. However, the difference between the antioxidant groups was statistically insignificant (Table 2).

**Table 1:** Inter-group comparison of level of significance. *Not significant; **Significant

Inter-group comparison	p-value
I vs IIA	>0.99 *
I vs IIB	0.737*
I vs IIIA	0.493*
I vs IIIB	>0.99*
I vs IVA	0.006 **
I vs IVB	0.201*
IIA vs IIB	0.522*
IIA vs IIIA	0.062*
IIA vs IIIB	0.995*
IIA vs IVA	<0.001**
IIA vs IVB	0.072*
IIB vs IIIA	0.022**
IIB vs IIIB	0.480*
IIB vs IVA	<0.001**
IIB vs IVB	0.015**
IIIA vs IIIB	0.389*
IIIA vs IVA	0.066*
IIIA vs IVB	0.888*
IIIB vs IVA	0.002**
IIIB vs IVB	0.172*
IVA vs IVB	0.941*

**Table 2:** Inter-group comparison of mean shear bond strength (MPa).

Group	Mean (MPa)	Standard deviation
I	14.61	3.72
IIA	14.88	0.91
IIB	16.86	2.79
IIIA	12.01	2.12
IIIB	14.36	2.29
IVA	8.89	1.65
IVB	10.28	3.55

## Discussion

Our results proved a decrease in SBS of composite resin to enamel immediately after bleaching, which was statistically significant when compared with the control group and the antioxidant groups.

Many studies have shown that the inclusion of peroxide ions may be reversed by the use of SA as an antioxidant [10-18]. Hence in this study, 10% solution of SA with application time of 10 min was preferred to be adequate for clinical application of the antioxidant in solution form.

 Etched appearance of enamel surfaces after ascorbic acid usage in bleached enamel specimens demonstrated super etching of the already bleached enamel surface under scanning electron microscope (SEM) [19]. Despite the wide-spread application of SA as an antioxidant, Savadi et al. [20] showed that the cumulative effect of SA and bleached material may lead to increased retention of pathogenic microorganisms in enamel surfaces. Usage of flavonoid-rich CB with proanthocyandinins as antioxidants presents as a viable alternative.

Treatment with 6% CB solution enhanced the bond strength of bleached enamel when compared to the bleached group not treated with any antioxidants, both when bonding was performed immediately and after storage in distilled water for two weeks following bleaching .

Both 6% CB solution and 10% SA solutions were found to be effective antioxidants with latter being more effective than the former but not statistically significant.

In Group IVB, the bond strength was higher compared to Group IVA. This could be attributed to the partial loss of oxygen diffusion layer over time at the tooth and composite interface [21]. Group II showed the highest bond strength among the study groups; both when bonding was performed immediately as well as after two weeks. Group III showed increased bond strength compared to Group IV; it had lesser bond strength than Group II and Group I.

Cranberry with proanthocyanidin with molecular weight ranging from 500 to 3000 g/mol had lesser penetration and bond strength when compared to SA with molecular weight 198.11 g/mol.

Moreover, different findings might be due to different antioxidant application methods. In previous studies [4, 22] during the 10-min treatment period the antioxidant solution was continuously refreshed and the enamel surface was agitated, which can enhance the antioxidant effect on the bleached enamel surface while in the present study the specimens were immersed in the antioxidant solution for 10 min as done by Lai et al. [3]. More recently a study conducted by Ismail et al. [18] concluded that an application of higher concentration of SA for a shorter time would reverse the negative effects of bleaching agents on bonding.

## Conclusions

In our findings 10% SA has proven to be superior compared to 6% CB solution in reversing the bond strength of composite resin to bleached enamel. We conclude that 6% CB solution can also be used as a viable antioxidant in the reversal of compromised bond strength of bleached enamel. Future studies are required to authenticate our observations.
